# Tungsten-Based Nanocatalysts: Research Progress and Future Prospects

**DOI:** 10.3390/molecules27154751

**Published:** 2022-07-25

**Authors:** Shaorou Ke, Xin Min, Yangai Liu, Ruiyu Mi, Xiaowen Wu, Zhaohui Huang, Minghao Fang

**Affiliations:** Beijing Key Laboratory of Materials Utilization of Nonmetallic Minerals and Solid Wasters, National Laboratory of Mineral Materials, School of Materials Science and Technology, China University of Geosciences (Beijing), Beijing 100083, China; shaorouke@163.com (S.K.); liuyang@cugb.edu.cn (Y.L.); miruiyu@163.com (R.M.); xwwu@cugb.edu.cn (X.W.); huang118@cugb.edu.cn (Z.H.); fmh@cugb.edu.cn (M.F.)

**Keywords:** tungsten, single-atom catalysts, nanocatalysts, tungsten compounds

## Abstract

The high price of noble metal resources limits its commercial application and stimulates the potential for developing new catalysts that can replace noble metal catalysts. Tungsten-based catalysts have become the most important substitutes for noble metal catalysts because of their rich resources, friendly environment, rich valence and better adsorption enthalpy. However, some challenges still hinder the development of tungsten-based catalysts, such as limited catalytic activity, instability, difficult recovery, and so on. At present, the focus of tungsten-based catalyst research is to develop a satisfactory material with high catalytic performance, excellent stability and green environmental protection, mainly including tungsten atomic catalysts, tungsten metal nanocatalysts, tungsten-based compound nanocatalysts, and so on. In this work, we first present the research status of these tungsten-based catalysts with different sizes, existing forms, and chemical compositions, and further provide a basis for future perspectives on tungsten-based catalysts.

## 1. Introduction

**Figure 1:**
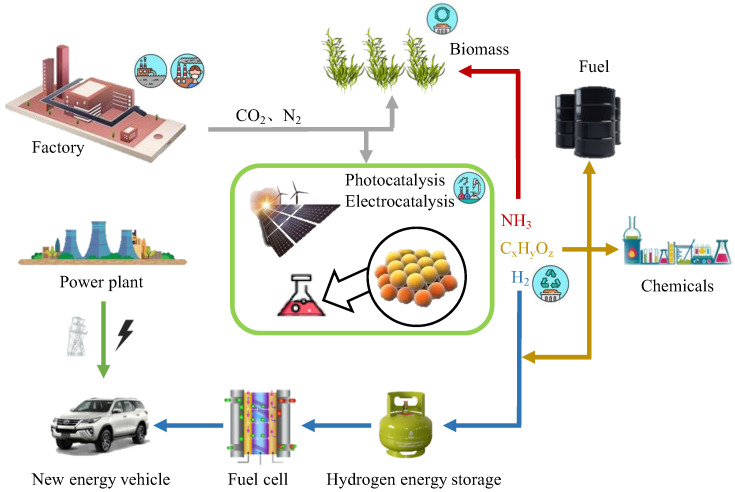
Illustration of future sustainable energy development.

In recent years, energy and environmental issues have been a part of the urgent needs of the world’s social and economic development, which both play a decisive role in the speed of this development. Therefore, saving energy and protecting the environment have become important issues for every country to speed up the construction of the green industry supply chain and to promote green development. At present, the research on energy mainly focuses on three aspects: energy generation, energy transportation and energy consumption. Currently, fossil fuels are commonly used, but they are a nonrenewable resource. With the increase in usage, fossil fuels are becoming gradually depleted. In addition, the low energy utilization rate of fossil fuels and serious environmental pollution have led to a crisis of energy and materials. Figure 1 shows a schematic diagram of possible sustainable energy development in the future [1]. Regarding the abovementioned information, the generation of energy is the most critical and effective step to solving the energy shortage. Researchers have mainly put forward three solutions to the energy generation process: (1) Increase the use of green energy (solar energy and hydrogen energy): electrolyze water through electrocatalysis technology and use sunlight to reduce CO_2_ through photocatalysis technology. (2) Extend the use of biomass energy. (3) Improve the utilization of low-chain alkanes, such as methane coupling, aromatization and propane dehydrogenation. However, regardless of the direction taken, accelerating the reaction rate, which means using catalysts with high performances, is key to improving energy efficiency.

In the field of catalysis, catalysts are the most important part, as they affect the rate and efficiency of the whole catalytic process. Noble metal platinum (Pt)-based catalysts are the most commonly used electrocatalysts with the best-known performances. They possess high specific surface area, many active sites, low overpotential (even close to 0 mV), and relatively small Tafel slope [2]. At the same time, they possess good catalysis for cathodic oxygen reduction reactions and anodic microscopic molecule oxidation reactions [3]. At present, the research on Pt-based catalysts mainly includes two aspects: (1) combining Pt nanoparticles with supports by loading or growth and (2) designing three-dimensional structures such as hollow, multi-hollow and spherical. In the research field of the first aspect, Han [4] supported Pt nanoparticles on nitrogen-doped carbon nanotubes. The research demonstrates that the catalyst that loaded 8.6 wt% Pt on the highest nitrogen-content graphite, has the best activity performance. Under acidic and alkaline conditions, the activity and stability of the hydrogen evolution reaction (HER) exceeded 40 wt% of the commercial Pt/C catalyst. On this basis, Wang et al. [5] doped trace iron (Fe) in Pt-loaded nitrogen-doped carbon nanotubes so that the catalyst had layered voids and a unique chemical composition. This kind of catalyst can be used not only in HER, but also in oxygen reduction reactions (ORR). The starting voltage is 918 mv, which is 117 mv higher than that of a commercial Pt/C catalyst under the same conditions. In the research field of the first aspect, according to the different crystal surfaces exposed by the catalyst, they retain different surface electronic structures, resulting in different catalytic properties [6]. In the second research field, the catalyst core-shell is an effective measure to improve the utilization of Pt, and there have been more and more studies on these catalysts recently. The most representative is the palladium (Pd) @ Pt catalyst prepared by Zhang [7]. The nanosize of the synthesized catalyst is uniform and microscopic. This kind of modified shell can effectively protect the Pd core, and the core can also regulate the electronic structure of Pt as the outer layer. Bai [8] further explored a kind of composite material with a new multifunctional core-shell structure. On the basis of giving the functionality of the composite material, through this double shell of a sandwich structure, it can not only inhibit the aggregation of noble metals but can also improve the stability of the catalyst. Pt-based catalysts are catalysts with excellent performance at present. In order to improve the reaction activity, the reaction conditions will be optimized first. The change of reaction condition may lead to the aggregation of the catalyst, and then the activity of the catalyst may decrease with the progression of the reaction [9]. At the same time, Pt resources are scarce on Earth and may not meet the commercial development of catalysts. Therefore, to find a catalyst with great performance and rich resources may retain great practical significance.

**Figure 2:**
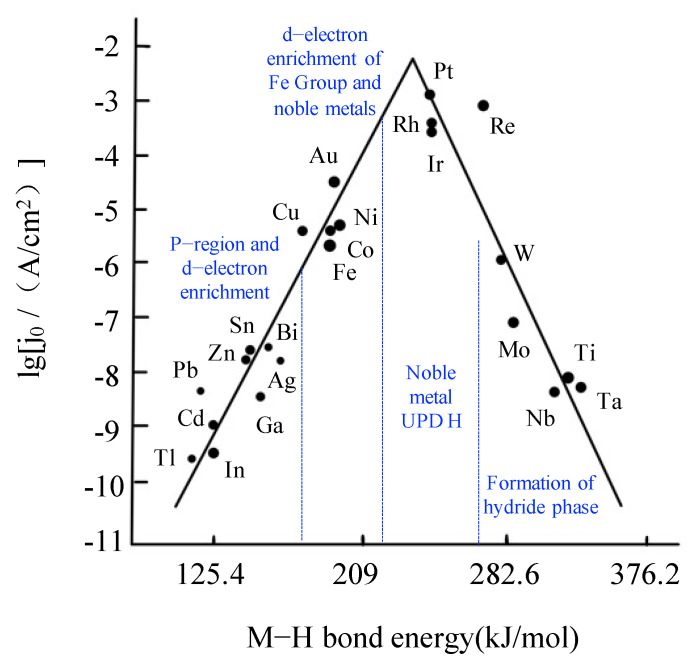
Volcanic formation relationship between exchange current density and M-H bond strength of the hydrogen evolution reaction on different metals.

As displayed in Figure 2, the relationship between the exchange current density (j_0_) and the bond energy (this value corresponds to the adsorption enthalpy of hydrogen atoms on metals) between different metals and hydrogen atoms in HER is shown, which is also known as a volcanic diagram. Due to the adsorption phenomenon on the active site of the reaction, when the adsorption enthalpy is microscopic, the adsorption will become the main step of controlling the reaction, resulting in a low reaction rate; when the adsorption enthalpy is too high, the intermediate adsorbent will be stably adsorbed on the surface, resulting in a low reaction rate. If we want to obtain the fastest reaction, intermediate adsorbent should have medium-adsorption enthalpy. Conversely, the adsorption enthalpy is mainly formed by the electrons of the H atom and the unpaired d electrons in the metal, which means that only the transition metal can significantly adsorb hydrogen. Combined with the data in Figure 2, it is considered that transition metals can be used in the catalytic field instead of noble metals. Figure 2 shows one of the important factors of reaction kinetics, because on the electrode surface, in addition to the interaction between the adsorbed H atom and the electrode, it is also affected by other conditions such as the solution; thus, the specific catalytic performance needs to be calculated.

**Figure 3:**
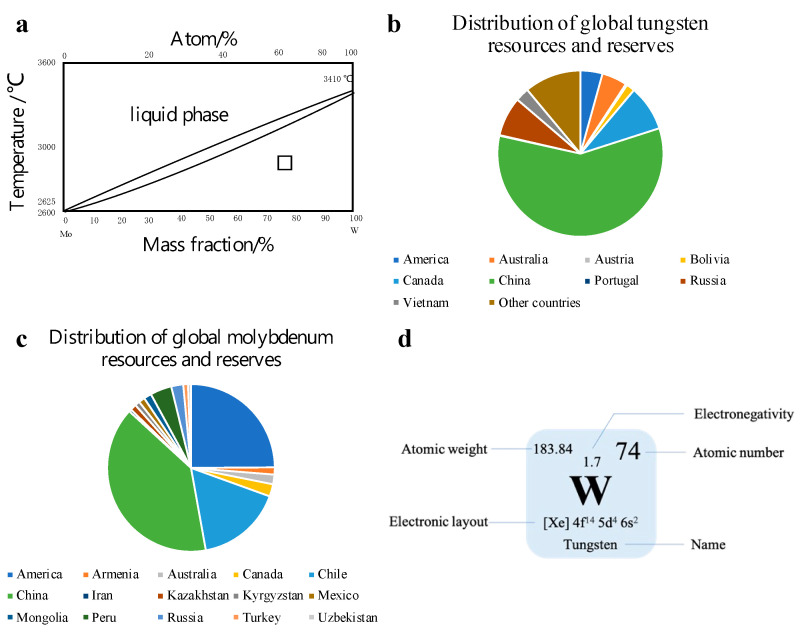
Advantages of tungsten-based catalyst. (**a**) State diagram of W and Mo. (**b**) Distribution of global tungsten resources and reserves. (**c**) Distribution of global molybdenum resources and reserves. (**d**) Basic data of tungsten.

In transition metals, tungsten (W) and molybdenum (Mo) are commonly used as catalysts. W and Mo are homologous elements with the same atomic radius, belonging to a body-centered cubic lattice. Furthermore, the lattice constants are almost the same [10]. Figure 3a demonstrates the state diagram of the two components. Through the curve, it can be observed that the solidus and liquidus of W and Mo are close. Hollak et al. [11] through research found that W has better selectivity than Mo, which can help W-based catalysts have better functionality. The average content of W in the Earth’s crust (0.001%) is higher than that of Mo (0.00011%). Compare with molybdenum resources, China has a relatively large proportion of tungsten resources in the world (Figure 3b,c). At the same time, W (Figure 3d) is also an environmentally friendly material, which can be −2, −1, 0, +2, +3, +4, +5 and +6 in chemistry. It is one of the most complex elements in transition metal elements [12], and it is a potential research object.

In the field of catalysis, in addition to selecting excellent elements, it is also important to understand the reaction principle and process, such that a closed-loop study of theory-synthesis-characterization has formed by testing the catalysts and by further understanding the theory according to the actual demand and then by regulating the synthesis steps of the catalysts.

In electrocatalysis, HER is an important semi-reaction of electrocatalysis for electrolyzing water, with two electron transfers. The reaction includes a Volmer step (discharge reaction), and the subsequent reaction may be a Tafel step (composite reaction) or Heyrovsky step (electrochemical desorption reaction) [13]. This means that there exist a Volmer–Tafel mechanism and a Volmer–Heyrovsky mechanism (* indicates the surface-active site of the catalyst):

(1) $$\mathrm{Volmer}\;\mathrm{step}:H^{+}+e^{-}+*\to *H;\;$$

(2) $$\mathrm{Tafel}\;\mathrm{step}:*H+H^{+}+e^{-}\to H_{2}+*;$$

(3) $$\mathrm{Volmer}\;\mathrm{Heyrovsky}:2*H\to H_{2}+2*.$$

Compared with HER, the hydrogen oxidation reaction (HOR) involves the same reaction steps, except for that the reaction is opposite. Oxygen precipitation reaction (OER) (2$H_{2}O\to O_{2}+4H^{+}+4e^{-}$) is another important semi-reaction in electrolytic water. The kinetic complexity of OER means that people have not fully understood it at the atomic level, but it is generally believed that OER is a process with four electrons that cooperate with a proton electron transfer reaction [13]; thus, the kinetic process of this kind of reaction is relatively slow. Only a large overpotential can accelerate its reaction rate. Compared with OER, ORR involves the same reaction steps, except for that the reaction is opposite. Although these reactions involve different reaction intermediates, mechanisms and electron transfer quantities, the description of evaluating activity and selectivity of these reactions still conform to the volcanic diagram, which is an important step for a rapid understanding of the principle of electrocatalysts.

In addition to the above reactions involving HER, HOR, OER and ORR, there are many other emerging electrocatalytic reactions, such as nitrogen reduction (NRR), H_2_O_2_ production, CO_2_ electroreduction, etc. NRR is a competitive reaction of HER: under certain conditions it initiates electroreduction ($N_{2}+6H^{+}+6e^{-}\to 2{\mathrm{NH}}_{3}$), which involves multiple intermediates. The reason for the production of H_2_O_2_ is to reduce oxygen to hydrogen peroxide ($O_{2}+2H^{+}+2e^{-}\to H_{2}O_{2}$). It is a process with two electron transfer processes; thus, the complexity is similar to HER. CO_2_ electroreduction is the same as ORR. It is a process with a multi-electron reduction reaction involving multiple reactions, but the products (water or hydrogen peroxide, also includes other possible CO_2_ reduction products) are different.

Among them, HER and OER are the most studied and often appear together. They are two half reactions in the electrolytic water reaction. Direct current is applied to the electrolyte, and then water as a solvent in the electrolyte undergoes electrolytic reaction. The H^+^ produced by electrolytic water migrates to the cathode, and the OH^-^ produced migrates to the anode; thus, the H^+^ near the cathode obtains electrons on the cathode, producing hydrogen (HER), and the OH^-^ at the anode loses electrons to produce oxygen (OER). In practical research, overpotential is usually calculated, because in an electrochemical reaction, the electrode potential will deviate from the equilibrium potential when the current flows through the electrode, further leading to polarization and making the actual electrolytic voltage greater than the theoretical electrolytic voltage. Usually, the potential difference between the reduction potential determined by the half reaction under thermodynamic conditions and the potential of the oxidation reduction reaction observed in the experiment is defined as overpotential. In addition to the influence of overpotential on the electrolytic voltage, current density, temperature and electrolyte type will also affect the electrolytic voltage, because they will affect the polarization of electrochemistry and further affect the electrolytic voltage.

The field of catalysis, in addition to electrocatalysis, includes photocatalysis and other catalyses. Photocatalysis produces electron hole pairs through the irradiation of sunlight, so as to produce strong redox potential and achieve the goal of purifying pollutants and synthesizing substances. Semiconductor material is commonly used. The smaller the band gap, the easier the electron transition and the higher the utilization of solar energy. However, solar energy is considered as a free and infinitely renewable clean energy; it still has not been widely used because of its high collection cost large floor area and unpredictability of solar energy [14].

By understanding the principles of electrocatalysis, photocatalysis and other catalyses, selecting excellent elements according to the theory becomes much easier. Scientific researchers have studied W-based catalysts and have considered them to be better potential research objects in transition metals. The most representative research is that Chen et al. [15] found that the catalytic performance of a tungsten-based catalyst is close to that of a Pt-based catalyst through electrochemical analysis. It was found that when W is loaded onto the carrier material, the band gap of the semiconductor will narrow, providing a large number of active sites, further improving the catalytic activity and stability. Meanwhile, Tu et al. [16] also found that W plays the role of “adhesive” between Pt and Cu, enhancing the bonding between Pt and Cu. This conclusion also opens up new opportunities for the preparation and application of efficient and stable tungsten-based nanocatalysts. Because the existence of a heterostructure will improve the catalytic activity, scholars have been studying the tungsten-based heterogeneous phase. Kou et al. [17] initially studied the interface effect of W. They successfully prepared tungsten carbonitride (WCN) twin nanocrystals. After characterization, it was found that WC and WN were chemically bonded at the molecular level, which gradually improved the catalytic activity and stability of the catalyst. These studies provide new opportunities for tungsten-based catalysts.

**Figure 4:**
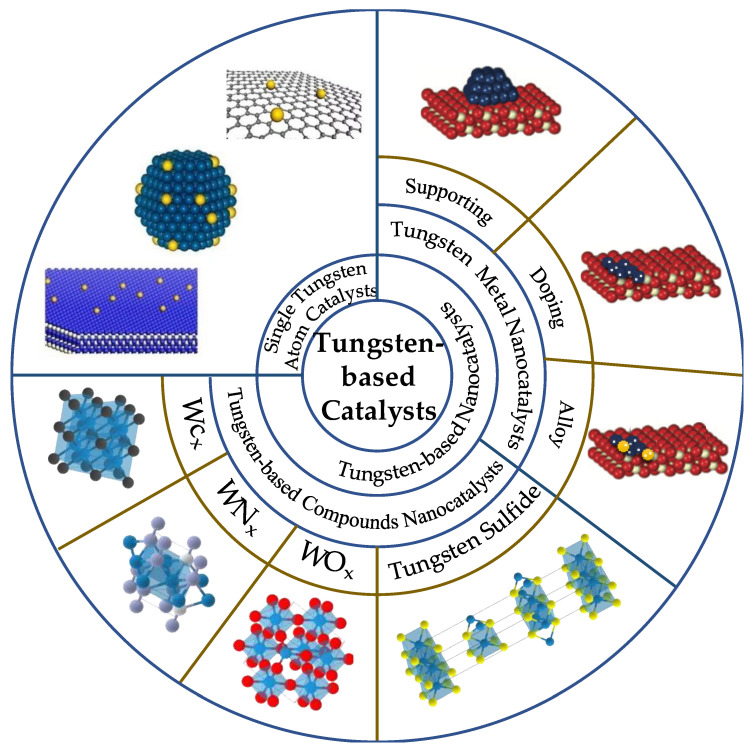
Learning direction of this paper.

Although tungsten-based catalysts are efficiently used in the industry with a variety of reactions, the research is still limited. Because tungsten-based catalysts are difficult to recover, with more and more relevant reviews published in the past [18,19], the direction and content of tungsten-based catalysts have been extended. These include tungsten monomers and tungsten compounds. In this paper, the single atom catalysts, tungsten metal nanocatalysts and tungsten-based compounds nanocatalysts are comprehensively reviewed and expanded in detail (Figure 4).

## 2. Single Tungsten Atom Catalysts

**Figure 5:**
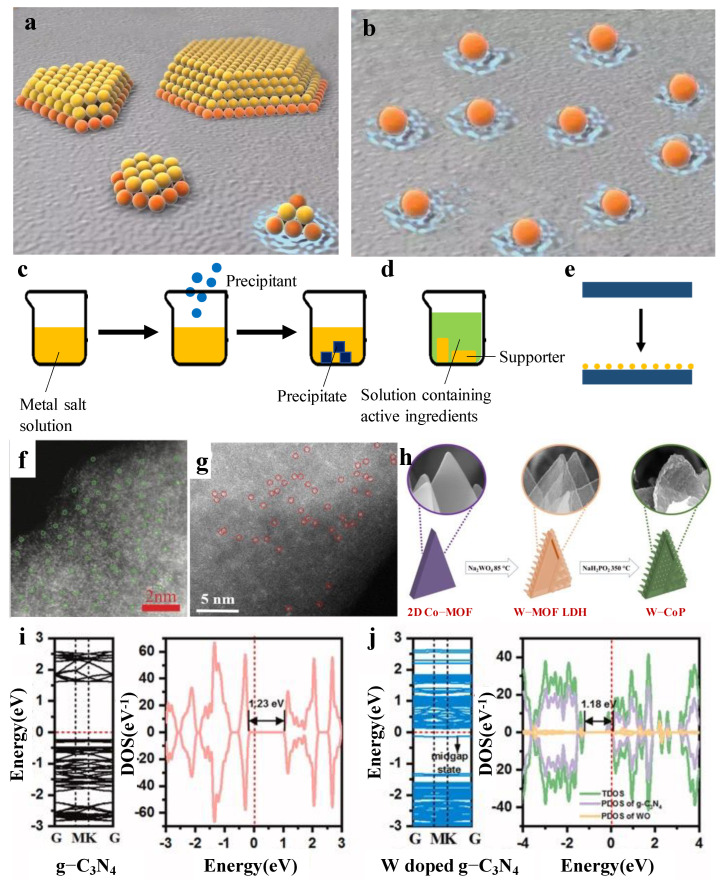
(**a**) Structural diagram of nanocatalysts. (**b**) Structural diagram of SACs. Diagram of the preparation method: (**c**) coprecipitation; (**d**) immersion; (**e**) atomic layer deposition. (**f**) Illustration of the formation of W-SAC. (**g**) HAADF-STEM image of the W-SAC. (**h**) HAADF-STEM image of the W-N-C catalysts. Band structures, total and projected density of states for (**i**) pure g-C_3_N_4_ and (**j**) W-doped g-C_3_N_4_.

In recent years, there has been an increasing amount of literature on single-atom catalysts (SACs). The first serious discussions and analyses of SACs had been discovered by Qiao [20]. SACs are defined as single or isolated metal atoms with catalytic activity anchored on the support that forms a composite catalyst with catalytic activity, which is a forward direction in the field of heterogeneous catalysis. Compared to nanocatalysts, SACs have the advantage of more excellent metal–support interactions [21], larger surface-free energy, higher selectivity, lower usage and so on, due to its particle size. As is displayed in Figure 5a,b [22], the orange particles are both connected with the support and the reactant. On the contrary, the yellow particles only touch the reactant, which indicates that SAC’s utilization is much better than the nanocatalysts. Herein, Xu et al. [23] found that the catalytic activity of SACs are highly correlated with the local environment of the metal center. Meanwhile, Li et al. [24] reported that the existence of a single atom could reduce the reaction energy barrier and weaken the competitive reaction adsorption, so as to promote the catalytic reaction. The above research provides a specific direction for the later research on SACs. The results show that [25] the microstructure, such as size effect, support effect, coordination effect and electronic effect, play an important role in catalytic performance.

Size effect is one of the most major influences in catalytic performance. The most representative is a series of Pt-based catalysts with different sizes prepared by Cheng et al. [26] through atomic layer deposition. By comparing the catalytic activities of nanoparticles, subnanoparticles and single atoms, it is proven that single Pt catalysts show the best catalytic performance, and the activity is 37 times that of commercial Pt/C. In terms of support effect, the carrier plays a decisive role. It not only provides a region for anchoring active species but also optimizes the local geometric and electronic structure of active metals. It is important to adjust the relationship between the metal carrier interaction and the catalytic performance.

The coordination effect describes differences in an adsorbate’s coordination with surface metal atoms due to different crystal facets and defects of support. Relevant theories show that the coordination environment of a single metal atom will change the adsorption affinity of the metal center, thus changing the catalytic performance. The most representative is the research conducted by Jakub et al. [27], who explored the difference of adsorption behavior of CO on a single iridium (Ir) atom under different coordination environments on the Fe_3_O_4_(001) surface.

As for the influence of the electronic effect on catalytic performance, the most typical catalyst is a catalyst composed of dispersed monatomic Fe reported by Gu et al. [28]. The research shows that Fe^3+^ has higher activity than traditional Fe^2+^. All the above studies provide theoretical guidance to improve the activity of SACs and to open up new opportunities.

Inspired by this, it was found that there are two main catalytic modes of single tungsten atom catalysts. There are common tungsten single atom catalysts; conversely, tungsten can be used as a single atom to optimize other catalysts; that is, it plays the role of a co-catalyst. The following are expanded from these two aspects.

### 2.1. Tungsten Single Atom Catalysts

SACs were studied to achieve application in many fields, such as HER, OER, ORR and photocatalysis by using coprecipitation, immersion, atomic layer deposition, etc. [29,30,31] (Figure 5c–e). With different types of supports, different properties are shown. The typical supports include metal organic framework (MOF) and carbon material, to name a few. Therefore, the central task is to understand the principle of the structures with atomic-level precision and then design excellent SACs to promote the catalysts’ properties. Electrochemical parameter of single tungsten atom catalysts currently reported are exhibited in Table 1.

#### 2.1.1. Tungsten Single Atom on Metal Organic Framework

Metal organic frameworks (MOFs), also known as porous coordination polymers (PCPs), are a kind of crystalline composite formed by the connection of metal centers or metal clusters with organic ligands in the form of coordination bonds. Compared with most microporous materials, MOFs hold better performance, and the specific surface area of MOFs is larger. At the same time, its structure and porosity can also be adjusted and controlled with different metals or ligands to realize functional modification.

Among single tungsten atom catalysts, the most representative research using MOF as a carrier was performed by Chen et al. [15]. They calculated the ΔG_H*_ of their W-SAC (a single W atom is supported on a derived N-doped carbon by pyrolysis strategy). An excellent electrocatalyst for HER usually gives a ΔG_H*_ value close to 0 eV. The results revealed that the W-SAC has a low ΔG_H*_ value of only 0.033 eV. In comparison, WC and WN possess a much higher ΔG_H*_ of −0.290 and −0.246 eV, respectively. These results suggested that the W-SAC was more of an advantage to HER than other W species. The study by Chen et al. [15] is the first example of single W atom catalysts. The obtained W-SAC exhibits high electrochemical HER performance under both alkaline and acidic media. Under a 0.1 m KOH solution, a low overpotential of 85 mV at a current density of 10 mA/cm^2^ was shown, which was close to that of commercial Pt/C. In this kind of solution, the Tafel slope was calculated to be 53 mV/dec, only 5 mV higher than that of commercial Pt/C. However, a low overpotential of 105 mV at a current density of 10 mA/cm^2^ and a microscopic Tafel slope of 58 mV/dec (in 0.5 m H_2_SO_4_ solution) was calculated. It is easy to find that single atoms were observed in HRTEM, mainly because amine groups in derived N-doped carbons prevent the aggregation of W species, making single tungsten atoms much easier to form. On this basis, further research was conducted by Jiang et al. [32]. They prepared W-SACs by loading tungsten onto zeolite imidazoline frameworks (ZIFs). The results showed that without amine groups, clusters can be observed (Figure 5f,g).

#### 2.1.2. Tungsten Single Atom on Carbon Materials

Hu et al. [36] found that the combination of the active center of the transition metal atom and the carbon support will give the transition metal catalysts excellent characteristics. Common carbon materials include graphene and fullerene. Graphene is a two-dimensional carbon nanomaterial with a hexagonal honeycomb lattice composed of carbon atoms with sp^2^ hybrid orbitals. It has the characteristics of simple preparation, good biocompatibility, excellent electronic transmission performance, and so on. Fullerene is similar to graphite in structure, which is a hollow molecule completely composed of carbon. Fullerenes have been widely used in catalysts in recent years [37] because of their excellent chemical properties, such as thermal stability and high hydrogen storage capacity. In recent years, carbon material has attracted more and more attention and is one of the more excellent supports. When the dispersed metal atoms are firmly anchored on the surface of carbon material, such electrocatalysts will show ultra-high specific surface area and excellent electronic performance [38], which provide great potential for innovation in the field of electrocatalysis.

The first to report the application of single-atom tungsten supported on graphene in the NRR was Gu et al. [33], based on the theory that the tungsten atoms have high NRR activity and selectivity for N_2_ fixation [39]. Gu et al. fabricate tungsten on graphene oxide (GO) through resin-chelating and self-template method. The reason for choosing GO as the support is that the introduction of O coordination weakens the W-N bond strength, which is a benefit for NRR. After DFT, the results showed that the single tungsten atoms are the origin of activity of the high NRR activity, which provides a theoretical basis for the application of single tungsten atom catalysts in NRR.

In comparison with the prevalent 2D material-supported SACs, the design of SACs is still challenging. Li et al. [40] introduced a new type of SAC, which a recently identified all-boron fullerene (B_40_) employed as the support. Among a series of candidates, the single tungsten atom supported B_40_ is screened out as the most feasible catalyst for the NRR with a low overpotential and high selectivity to passivate the competitive hydrogen evolution process. These results offer a promising NRR electrocatalyst and a hopeful pathway for a performance enhancement strategy.

### 2.2. Tungsten Single Atom Co-Catalysts

Tungsten is also widely used as a single atom to optimize other catalysts. The most representative work in HER is performed by Wu et al. [34]. Their work focused on preparing another kind of W-SAC, in which the support is cobalt phosphide (CoP). W-CoP was prepared by etching and phosphating (Figure 5h). First, in 1.0 M KOH, it showed the overpotentials of 40 mV to realize 10 mA/cm^2^ and reached a smaller Tafel slope of 47 mV/dec. Conversely, a low overpotential of 48 mV at a current density of 10 mA/cm^2^ and a microscopic Tafel slope of 56 mV/dec (in 0.5 m H_2_SO_4_ solution) was calculated. Compared with other W-SACs, the surface of W-CoP is coarser because of the 3D shape of the CoP. It is speculated that there may be more surface-active sites. The above study provides a research direction about single tungsten used as a co-catalyst.

Developing a kind of electrocatalyst toward HER/OER is crucial for the spread of hydrogen energy industrialization. Herein, Wang et al. [35] prepared a W single-atom-doped heterostructure grown on nickel foam (NF), denoted as W-NiS_0.5_Se_0.5_. The HER performances were tested in 1.0 M KOH, showing that W-NiS_0.5_Se_0.5_ exhibits a lower overpotential of 39 mV to reach 10 mA/cm^2^, which is comparable to commercial Pt/C (36 mV). Furthermore, W-NiS_0.5_Se_0.5_ shows a Tafel slope of 51 mV/dec and a turnover frequency (TOF) of 1.105/s at −100 mV (RHE), which is approximate to commercial Pt/C (36 mV/dec) and higher than commercial Pt/C (0.222/s), respectively. In OER, the LSV curves showed that W-NiS_0.5_Se_0.5_ exhibited a lower overpotential (171 M V) at 10 mA/cm^2^ than that of IrO_2_ on NF (337 mV). Furthermore, W-NiS_0.5_Se_0.5_ shows a Tafel slope of 41 mV/dec and a turnover frequency (TOF) of 1.85/s at −250 mV (RHE), which is smaller than those of IrO_2_ on NF (92 mV/dec) and better than IrO_2_ on NF 0.0017/s. Finally, W-NiS_0.5_Se_0.5_ is expected to assemble a two-electrode water splitting system by using it as a cathode and anode, opening the application direction of the tungsten single atom as a co-catalyst of bifunctional catalysts.

Tungsten single atom as a co-catalyst cannot only be used in electrocatalysis, but can also be used in photocatalysis. Photocatalysis is considered as a promising strategy to produce hydrogen. The most representative research is performed by Zhang et al. [41]. A single W atom-dispersed graphitic carbon nitride (g-C_3_N_4_) was prepared in combination with a thermal oxidation etching process. Importantly, no significant clusters could be observed in the HAADF-STEM images, which suggests that a single W atom appeared. Meanwhile, the Brunauer–Emmett–Teller (BET) results show that the mesoporous structure was generated, which usually renders more active sites, had a higher mass transfer efficiency, and was favorable for improving the photocatalytic activity. Based on the results of the DFT calculations, it can be concluded that the bandgap of the W-doped g-C_3_N_4_ decreases compared to the pure g-C_3_N_4_ through density of states (DOS) results (Figure 5i,j). This work paves an efficient path toward a high-loading W-SAC and opens new insight into its application.

On this basis, Gu et al. [42] further innovated a novel kind of W-SAC with a single W atom supported on g-C_3_N_4_, compared with the study by Zhang et al., which was focused on for a one-step calcination of mixed precursors, and this method is more convenient. The results obtained are roughly consistent with those of their predecessors. They also reported that single W atoms broaden the visible-light absorption of pure CN. The bandgap energy from 2.66 eV (pure CN) reduces to 2.47 eV (W-CN).

### 2.3. Summary

Atomic tungsten is the origin of catalyst activity instead of other W-based species; thus, W-SACs have broad prospects. However, there are still many deficiencies. When the metal particles are reduced to the single atomic level, the specific surface area will increase sharply, resulting in an increase in metal surface free energy. It is easy to agglomerate and form large clusters during preparation and reaction, resulting in the deactivation of the catalysts. Therefore, the stability and load of SACs are great challenges. To overcome these problems, many strategies have been proposed, including defect engineering and carrier selection. At present, choosing an appropriate support is the most effective way. Zhang et al. [43] explored a new type of monatomic layer cluster for advanced catalytic fields. Similarly to SACs, this kind of catalytic material is still in the early stages of research, but it may still bring greater opportunities and challenges to the catalytic field in the future.

## 3. Tungsten Metal Nanocatalysts

**Figure 6:**
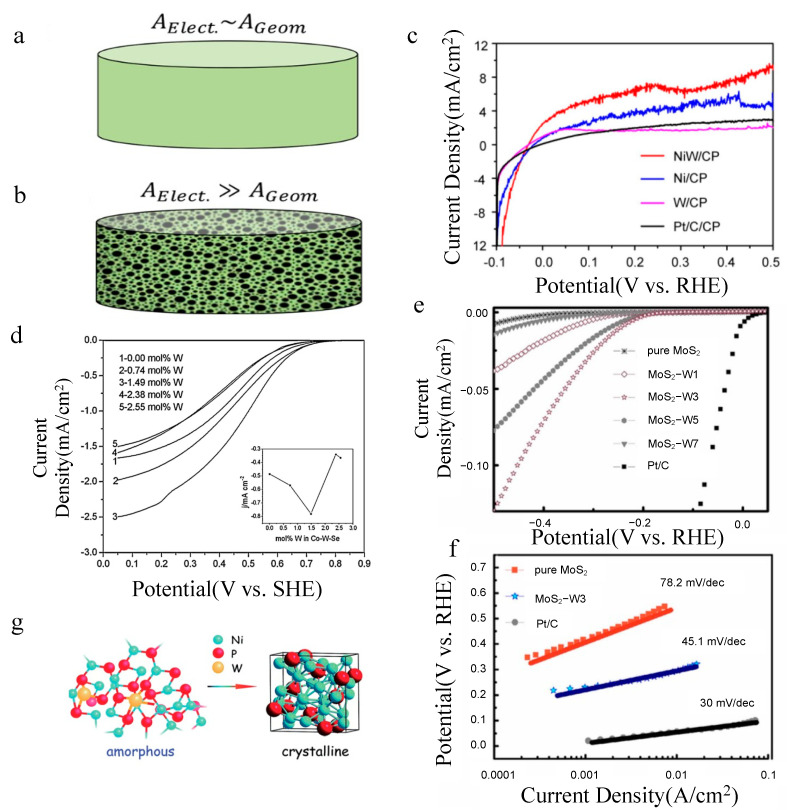
(**a**) Schematic diagram of conventional and (**b**) nanostructured catalysts. (**c**) Polarization curves of the Co–W–Se catalysts toward ORR and a plot of the corresponding kinetic current density j_k_ at 0.5 V (versus SHE) versus W molar ratio. (**d**) Experimental linear sweep voltammetry curves of the samples. (**e**) Tafel plots MoS_2_-W3 and pure MoS_2_. (**f**) Scheme of the structure transformation after heat treatment. (**g**) HOR catalytic performances of NiW/CP, Ni/CP, W/CP, Pt/C/CP in H_2_-saturated 0.1 M KOH electrolytes by LSV measurements.

As another kind of catalyst, nanoparticle catalysts have been widely used in industrial production and have a longer development history than that of SACs. Compared to SACs, nanoparticles loaded on the supports will hold a large specific surface area and many electrochemical active sites; thus, the electrochemical active area is not the only area in contact with the electrolyte. As shown in Figure 6a,b [44], the geometric area of traditional electrodes and nanostructured electrodes is compared with the electrochemical active area. When the size of the catalyst decreases to a nano size, the surface effect, volume effect and quantum size effect will be shown. This kind of special property is becoming a hot spot in the 21st century.

During the past years, many more applications have become available on electrolytic water, photocatalysis, low-chain alkanes and so on. At the same time, the supports of them are in varied forms, from carbon to mesoporous materials, with each of them showing different excellent performances. Carbon materials, especially carbon nanotubes (CNTs) and graphene, exhibit a large π bond, which has good conductivity. Because of its high theoretical specific surface area and good strength, it has immediately become a popular material in the field of catalysts. In addition to the above carbon materials, biochar also has the potential to become a widely used support. It has a wide range of resources and low cast. In addition, biochar has a strong cross-linking effect with metal nanoparticles before the reaction and high defect sites in the reaction [45]. Therefore, biochar has recently been widely studied by researchers [46,47,48]. Mesoporous is another kind of support, it possesses the characteristics of high specific surface area, regular and orderly pore structure, narrow pore size distribution and continuous adjustable pore size, which give it a role in catalytic reactions. In addition to SiO_2_, the common mesoporous nickel foam and the self-made mesoporous have also been studied [49,50].

Due to the characteristics of adjustable components and reconfigurable electronic structure, nanocatalysts show broad development prospects in the field of catalysis. This section is based on the review of nanocatalysts with metallic tungsten as the active component and summarizes the existence form (including supporting, doping and alloy) of nanometallic tungsten in nanocatalysts.

### 3.1. Supported Tungsten Metal Nanocatalysts

Supported catalysts are catalytic active components that are anchored on the surface of the support. The support is mainly used to support the active components; thus, the catalysts have specific physical properties, while the support itself generally does not exhibit catalytic activity. Here, tungsten represents the catalytic active components.

Because it is difficult for a metallic simple substance to exist in chemical reactions, there are little research results regarding metallic tungsten nanoparticles. The most representative research was performed by Wang et al. [51]. They prepared a tungsten nanowire array on SiO_2_ substrates through Ni-assisted CVD. With the results of SEM, EDS and XRD, they found that the tip of the nanowire was Ni_4_W, while the stem of the nanowire was W. In this work, they inferred W generated only by replacing the bare SiO_2_ substrate with the Ni-coated SiO_2_ substrate. The orientation relationship between the catalyst particles and the nanowire is expected for other nanowires, providing a framework for further research.

### 3.2. Tungsten Alloy Nanocatalysts

At present, alloys hold broad application prospects in heterogeneous catalysis due to their various catalytic active sites produced by their vast element combinations and complex geometric structures [52]. Alloying Pt with transition metal elements is an effective way to obtain high catalytic performance. However, the alloys are prone to electrochemical leaching and dissolution at andic potential, which devastates the stability of the catalysts. In recent years, Pt_x_Cu_y_ alloy has been confirmed to exhibit high catalyst performance [53,54]. Therefore, finding out a technique to improve the stability is key. A nucleation-trapping strategy performed by Tu et al. [16] was found to be effective in improving stability. Tu et al. incorporated W atoms into the bulk of Pt_x_Cu_y_. The ORR polarization curves show that the half-wave potential for Pt_2_CuW_0.25_/C was 0.929 V, which is higher than Pt/C (0.891 V). In addition, its Tafel slope (55.8 mV/dec) is lower than that of Pt/C (63.4 mV/dec), showing better catalytic performance. It was important to confirm whether the stability had improved. The stability was evaluated via an accelerated durability test (ADT). After 30,000 cycles, the ECSA of Pt/C declined by 41.9% compared with the initial values. Conversely, the ECSA of Pt_2_CuW_0.25_/C increased by 4% compared with the initial values. This work demonstrates a unique perspective in designing high-performance catalysts by using doped tungsten alloys.

On this basis, Xiong et al. [55] studied the tungsten alloy in other fields, and they found that NiW performs well in HOR activity; furthermore, both thermodynamic and kinetic first-principle calculations demonstrated that the HOR performance of Ni (111) can be largely enhanced by W alloying. They prepared NiW grown in situ on carbon paper (NiW/CP), and compared the HOR activity of NiW/CP, Ni/CP, W/CP and Pt/C/CP. Through LSV measurements (Figure 6c), it was found that NiW/CP shows the best HOR activity among all samples.

The existing theory shows that the increase in three-dimensional channel size can effectively avoid the overlap of double electric layers on the catalyst surface [56], which provides a new way for the design of three-dimensional nanomaterials. Based on this, there is a large volume of published studies describing the role of 3D transition metal hydroxides [57,58], which helps in promoting water dissociation. The most representative research was performed by Zhang et al. [59], where they prepared the amorphous structure of CoW(OH)_x_ by the electrodeposition method with Co and W precursors onto a Ni foam substrate. Then, the electrocatalytic activity was assessed in 1.0 M PBS, with the results showing that it only requires overpotentials of −73.6 and −114.9 mV to reach the current densities of −10 and −20 mA/cm^2^, respectively. Furthermore, the CoW(OH)_x_ also has a low Tafel slope (149 mV/dec), as well as the lowest charge-transfer resistance (Rct) and largest ECSA among all samples. Moreover, the stability was shown to be good, because there is no obvious degradation observed after 10,000 cyclic voltammetry cycles. This study may provide a new strategy to design efficient electrocatalysts for HER in neutral environments.

In summary, the above study about tungsten alloy nanocatalysts confirms that tungsten-based alloys well retained structural rigidity, resulting in high catalytic performance and long-time structural stability in an electrochemical environment. These works demonstrate a new perspective to prepare excellent performance catalysts by simultaneously improving activity and stability.

### 3.3. Nanocatalysts Doped with Tungsten Atoms

Doped tungsten atoms are a common form in tungsten nanocatalysts. The principle is that tungsten ions enter the lattice vacancy or replace a little part of the support to form a compound structure, resulting in lattice structure defects and an increase in the specific surface area so as to improve the adsorption capacity and adsorption rate [60]. Conversely, the tungsten is introduced to regulate the rate of water adsorption and for the dissociation steps [61]. Based on this idea, W is popular in water splitting, because the W has a strong affinity to water. Thus, there is a large volume of published studies describing the role of doping [62]. In this process, particle agglomeration and melt sintering are inhibited.

Zhao et al. [63] were the first group to study tungsten doping. They investigated the differential impact of tungsten content on CoSe_2_. Through the RDE polarization curves for oxygen reduction on the tungsten-doped Co-Se catalysts recorded at 1600 rpm and 10 mV/s in O_2_-saturated 0.5 M H_2_SO_4_, the results showed that the addition of tungsten enhances catalytic activity. The kinetic current density of the Co–W–Se catalysts at 0.5 V (versus SHE) varies with tungsten content, and the highest value at 1.49 mol% W is larger than that of the W-free Co–Se system (Figure 6d). Furthermore, the open circuit potentials (OCP) were also tested, as shown in Table 2. The OCP value of 1.49 mol% W (0.81 V) is higher than that of the W-free Co–Se catalyst, but lower than that of the commercial Pt (1.08 V). These results provide a theoretical possibility, opening up new opportunities for the development of tungsten atom doping.

On this basis, Huang et al. [64] optimized the carrier material and prepared a carbon layer-supported tungsten-doped vanadium nitride (VN) nanoparticle. Compared with its predecessors, the advantage of this kind of catalyst is that a VN carrier is used. VN has the function of refining grains, which can make the nanoparticles of W smaller and more uniform so as to improve the catalytic activity.

Except for the field of ORR, HER has also been studied widely. As a most common reaction in electrolytic water, the research about it is more advanced. Due to nanocatalysts with different properties, it can be obtained zero-dimensionally, one-dimensionally, two-dimensionally and three-dimensionally. The three-dimensional porous structure can provide a larger specific surface area and catalytic active sites [65] so as to obtain nanocatalysts with better performance.

On this basis, MoS_2_ is considered to be a better 2D support because of its low cost, high chemical stability and excellent electrocatalytic properties [66,67]. With respect to the issue, the structure design of MoS_2_ has attracted more interest because it obtains more active sites [68]. Therefore, He et al. [69] reported that replacing Mo with W atoms will induce a lattice distortion but will still retain a similar monolayered. They synthesized a 3D MoS_2_-W_x_ nanoflower structure via the hydrothermal method. The different contents of tungsten were also studied by them (MoS_2_-W1, MoS_2_-W3, MoS_2_-W5, MoS_2_-W7). Through SEM, the surface of pure MoS_2_ was smooth, with the content of tungsten increasing and the images becoming much rougher, which suggested that after doping W into MoS_2_, MoS_2_-W presents nanoparticle structures assembled by ultrathin flakes. Among them, MoS_2_-W3 shows the best morphology. Under BET, all samples were tested, and 3D MoS_2_-W3 possessed the richest microstructure and a relatively larger BET surface. Furthermore, the electrochemical HER tests were performed under the condition of a 0.5 M H_2_SO_4_ solution, using glassy carbon-coated catalysts as the working electrode. The results indicated that 3D MoS_2_-W3 is the most active catalyst among all samples, rendering a low onset potential at −0.20 V and large current density of 124 mA/cm^2^ at −0.5 V (Figure 6e), which is in agreement with the results of SEM and BET, demonstrating that the catalyst performance is related to pore size and specific surface area. However, it only proved that doping can help obtain more active sites and lead in higher HER performance. Through the polarization curves and Tafel slope, it is easy to find that there is still a gap with Pt/C (Figure 6f). Thus, there is a long way to go for improving HER performance.

In the field of HER, doping crystalline tungsten can help improve performance. In addition, since the doping of amorphous tungsten metal was reported for the first time [70], amorphous tungsten metal has been a research hot spot in the field of catalysis. Because of its special structure of long-range disorder and short-range order, this gives it a much higher catalytic activity than the corresponding crystalline catalysts [71]. Jin et al. [72] studied the crystalline structure and amorphous structure regarding HER performance because heat treatment can make amorphous tungsten transform into a crystalline structure (Figure 6g). Considering the decline in HER performance with increasing crystallinity, for the above two reasons, three-dimensional amorphous materials are often used as support.

To the best of our knowledge, Jin et al. [72] are the first to successfully fabricate amorphous tungsten-doped nickel phosphide (a-WNP) microspheres with three-dimensionally (3D) with a grain-mediated co-deposition method. A densely wrinkled surface is shown in the TEM results, indicating that the spheres possess a rough three-dimensional structure. Furthermore, the STEM images confirm that Ni, P and W are in the a-WNP microsphere in a homogenous distribution. The catalytic performance for a-WNP deposited on the Ni foam was investigated in 0.5 M H_2_SO_4_ by comparing it to Pt/C (20 wt%). The polarization curves reveal the onset potential of a-WNP at −50 mV, which is close to that of Pt/C of ~0. Moreover, the Tafel plots demonstrate that a-WNP has a microscopic Tafel slope of ~39 mV/dec, close to 30 mv/dec of Pt/C. The exchange current density for a-WNP was determined to be 4.4 × 10^−2^ mA/cm^2^ compared to 7.0 × 10^−1^ mA/cm^2^ for Pt/C, which has a desirable value among the nonnoble metal HER catalysts. Durability is another important property of catalysts, of which the authors also tested it by CV. After the sweep between +0.2 and −0.3 V for 1000 cycles, compared with the initial catalysts, there was still more than 95% activity remaining for a-WNP. Finally, the SEM of a-WNP before and after long-period catalysis reveals that a-WNP almost keeps its initial 3D ravine-like structure, which may extend into a specific area and into the active sites. Conversely, HER in alkaline solution was also investigated, and the polarization curves reflect an overpotential of 160 mV at 20 mA/cm^2^ for a-WNP. Although the performance declined compared with the acidic electrolyte, such high activity in 1.0 M KOH is still good for HER catalysts. Moreover, they also display a model of amorphous structure that exposes some unsaturated P sites bound with a tungsten dopant. According to the report, the presence of the unsaturated active sites would probably be helpful for HER [73]. The results promote a new route to gain both 3D and amorphous doped materials with special electrochemical properties. Perhaps in the near future, the doping of amorphous tungsten can be applied not only in HER, but also in other electrochemical reactions.

Furthermore, OER is a significant part of the field of catalysts. The most representative research has been performed by Nguyen et al. [74], in which they prepared an amorphous tungsten-doped cobalt oxide (W:CoO) via an electrochemical oxidation process. Among all the OER catalysts that have been reported, cobalt oxide is one of the most potential supports, because of its high activity and robustness [75]. Nguyen’s study shows that W:CoO requires an overpotential of 350 mV to reach a catalytic current density of 10 mA/cm^2^, even lower than that of IrO_2_ and RuO_2_ [76] and it has been compared so as to achieve status as the best cobalt-based OER catalyst. However, because CoO is harmful to water, it is deeply limited in its development.

Compared with CoO or other metal oxides, silicates combine the merits of being environmentally friendly, naturally abundant and chemically stable with ease [77]. On this basis, Zhu et al. [78] conducted another study, in which they designed a kind of tungsten-doped manganese silicate via thermal vapor deposition and a subsequent microwave reaction. Without the doped tungsten, the Tafel slope of the sample is 114.55 mV/dec, which is better than the Co-based materials at pH = 7 [79]. Specifically, when tungsten was doped into the MnSi, the Tafel slope (109.38 mV/dec) was similar to the sample without tungsten. Hence, the authors obtained a deeper study, the intrinsic catalysis of MnSi is better than MnSi-W, indicating that W doping does not contribute to intrinsic OER activity but instead plays an important role in enhancing the exposure of active sites and the durability of catalysts. Although the intrinsic activity of MnSi is better, the OER activity of MnSi-W is better because the intrinsic activity only shows the natural and physical chemistry of single active sites. Based on the above research, we found that the W doping into silicates combines differently from other types of doping, which directly alters the d-band center and changes the activation energy. In contrast, W doping into combined silicates increases the number of active sites and stabilizes the silicate. This research may provide a new theory about W doping in the OER field and has created a new market for OER.

Regardless of the type of catalyst reaction, the preparation of an efficacious and convenient catalyst is the main point. The most effective way to develop a catalyst is as a dual function. However, it is difficult to couple HER and OER catalysts in an integrated electrolyzer in the same electrolyte with good overall water-splitting performances because of their different stabilities and activities [80]. On this basis, Ren et al. [81] innovatively reported W-doped CoP nanoneedle arrays fabricated onto a flexible conductive carbon cloth as HER and OER electrocatalysts for overall water splitting in an alkaline electrolyte. The results showed that the sample had low overpotentials of 32 and 77 mV at a current density of 10 mA/cm^2^ in 0.5 M H_2_SO_4_ and a 1 M KOH and microscopic Tafel slope of 57 and 65 mV/dec, respectively. Furthermore, the sample also presented good OER activity with a low overpotential of 252 mV at a current density of 10 mA/cm^2^ and a microscopic Tafel slope of 74 mV/dec in 1 M KOH, respectively. Based on the above research, the authors exemplified a small cell voltage of 1.59 V to generate a current density of 10 mA/cm^2^, which is superior to many recently reported bifunctional electrocatalysts in overall water splitting. The results provide a new strategy to design and prepare a kind of catalyst with both HER and OER performances.

In addition to single metal doping, there is also bimetallic doping. Bimetallic doping is a polymetallic synergistic effect that refers to the performance produced by doping two or more metals, which is higher than any of its solitary components, such as higher catalytic activity or better stability. It is believed that the reason for the improvement in bimetallic properties comes from the mixing of more than one active metal, the generation of proton position, or the change in electronic structure caused by the change in the surface morphology of the nanoparticles. In the field of tungsten as one of the bimetals to doping, the most iconic of all is the research performed by Wu et al. [82]. Here, ultrathin tungsten-doped nickel iron-layered double hydroxide (Ni-Fe-W LDH) nanosheets were grown on nickel foam (NF) by a water bath reaction. When applied as OER catalysis, the best sample required 230 mV to drive a current density of 50 mV/cm^2^, and the Tafel slope was low (64 mV/dec). Meanwhile, when the ratio of W doping increased, the overpotential also increased, which might have been attributed to the introduction of W to create more electrochemically active sites [83]. In this work, the introduction of W helped to enhance conductivity, create favorable kinetics, and modify the electronic structure, producing good synergistic effects for catalysis and a general way to fabricate bimetallic and trimetallic W-based catalysts.

A comparison of catalytic activity of tungsten-doped nanocatalysts showed the expediency of doping. This is because W doping induces the re-arrangement of surface atoms and effectively increases the number of active sites [84]. However, there is still a long way to go to find out which materials should be doped and at what amounts. If these are studied, it will be a major breakthrough in the direction of doping to improve catalytic activity.

### 3.4. Summary

Electrochemical parameters of tungsten metal nanocatalysts currently reported has been exhibited in Table 3. The key objectives of nanocatalysts are to prepare catalysts with high selectivity, high activity, low energy consumption and that last a long time. In order to obtain nanocatalysts with better performance, more recent attention has focused on the provision of more active sites, the regulation of doping and the regulation of phase [85].

Supported tungsten nanocatalysts is a method to add more active sites. In order to provide more active sites, scientists also prepare nanoparticles with different morphologies or carrier materials with different shapes (one-dimensional, two-dimensional and three-dimensional) to expose more active sites. Doping is another common way to increase catalyst properties. The key is to understand the relationship between load particle and carrier. Thus, Deelen et al. [85] systematically introduced metal support interaction (MSI), including charge transfer, contact interface range, nanoparticle morphology, chemical composition and metal support strong interaction, which has a good guiding significance for the development of nanocatalysts in the future. On this basis, Wang et al. [86] studied the size effect of nanoparticles and the regulation of doping. Because nanosized catalysts have different effects on catalytic activity from two aspects, geometric structure and electronic structure, the development of nanocatalysts has always been limited, which is a major challenge in the field of heterogeneous catalysis. In terms of geometric structure, the smaller the size of the metal particles, the greater the exposure proportion of the atoms, and the more active sites there are for the catalytic materials. From the perspective of electronic structure, the size effect affects the electronic energy level of metal particles, and it will change the orbital hybridization and charge transfer between catalytic materials and reactants. Wang et al. [86] found that the geometric effect controls the performance of the dominant reaction on large (>4 nm) particles. On smaller (<4 nm) particles, the electronic effect controls the performance of the dominant reaction. This provides a guiding direction for the design of metal nanocatalysts with high activity and high selectivity, while it is seldom studied regarding adjusting the electronic structure by changing the phase of the materials [87]. It is difficult to understand the structure–property relationship of alloys. Therefore, there is a long way to go before developing a kind of catalyst with excellent performance.

## 4. Tungsten-Based Compounds Nanocatalysts

In the field of tungsten-based nanocatalysts, there are not only tungsten metal nanocatalysts, there are also tungsten compound nanocatalysts. Scholars have extensively researched the formation of these kinds of metal and nonmetallic transition materials due to their low resistivity and electronic stability [88]. Compared with tungsten metal nanocatalysts, the research on tungsten-based compound catalysts is more extensive, which brings new opportunities for the development of it. There are many methods to prepare nanocatalysts, mainly including the chemical reduction method, electrochemical method, and so on. Chemical reduction is the most common method. The preparation of nanocatalysts by this method is widely used in the laboratory because of its mild reaction conditions and low requirements for instruments. Metal salts are reduced to metal nanoparticles under certain conditions by using surfactants or other reducing agents. However, it is still difficult to obtain a microscopic size and uniform nanoparticles, due to the phenomenon of particle agglomeration in the calcination process. Zeng et al. [89] were the first to study this method. Pt/C nanoparticles were prepared by using citrate as the reducing agent. It was found that the selection of an appropriate reducing agent also affected the catalytic activity of the catalysts. On this basis, Komvokis et al. [90] compared the catalysts prepared by the chemical reduction method with the catalysts prepared by the impregnation method. The results showed that the average particle size of the nanocatalysts prepared by the chemical reduction method was 1–3 nm, which was much smaller than the average particle size (10–80 nm) of the nanocatalysts obtained by the impregnation method, and which showed greater dispersion. This provides a new opportunity for the preparation of nanocatalysts by chemical reduction. Electrochemical reduction is another kind of effective reduction method, which needs to provide external energy to the reaction system so that electrons can be transferred to reduce the metal ions. The most representative study is the metal nanocatalysts prepared by Zhou et al. [91] through the electrochemical method. It was found that the electrochemical method can control the shape synthesis, breaking through the limitations of the traditional chemical reduction method in that it can only synthesize low surface energy, and opening up a new prospect for the shape control synthesis of nanoparticle catalysts with high surface energy and high activity. Due to its energy consumption, further development of the electrochemical method is limited. In addition to the two preparation methods, there is chemical vapor deposition, the microwave-assisted method, and the template method [92,93,94]. Based on the above research, this paper introduces various components and structures of tungsten compounds (including tungsten carbide, tungsten nitride, tungsten oxide and chalcogenide) in detail below.

### 4.1. Tungsten Carbide

**Figure 7:**
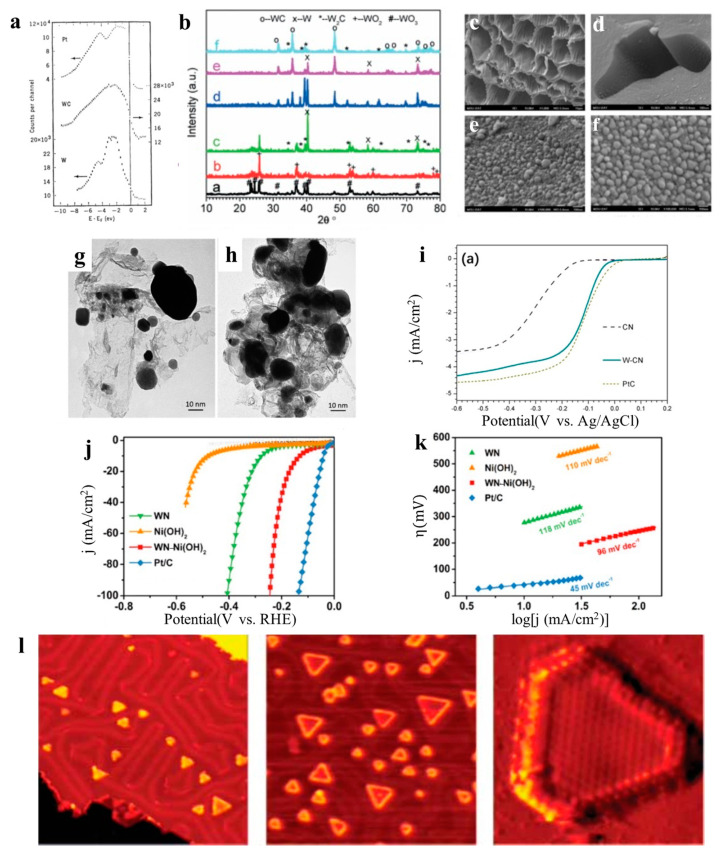
(**a**) Valence band XPS spectra of Pt, WC and W (111). (**b**) The XRD patterns of tungsten-promoted biochar samples prepared by carbothermal reduction for 1 h at different temperatures. Tungsten promoted the SEM image of biochar after carbothermal reduction at 1000 °C for 1 h (**c**–**e**) and carbothermal reduction at 1000 °C for 3 h (**f**). TEM images of tungsten-promoted biochar after carbothermal reduction for 1 h (**g**) and 3 h (**h**) at 1000 °C. (**i**) ORR polarization curves for CN, W-CN and PtC in 0.1 M KOH with a scan rate of 5 mV/s and a rotating rate of 1600 rpm. (**j**) LSV curves and (**k**) Tafel plots of WN-Ni(OH)_2_, WN, Ni(OH)_2_ and Pt/C. (**l**) Atomic structure of a nanotriangular transition metal sulfide in the monolayer.

As early as 1974, Bennett et al. [95] proved that near the Fermi level, the electron density of tungsten carbide (WC) is closer to that of Pt than that of W (Figure 7a). In addition, according to the performance of Pt, it was pointed out that the catalysts with better performance should hold the following two characteristics: (1) high Fermi level; (2) high magnetic susceptibility. Tungsten carbide has the above two advantages. After this, relevant studies have also confirmed [96] that tungsten carbide has catalytic behavior similar to Pt. In addition, tungsten carbide has been found to be used in hydrogenation reactions, hydrocarbon isomerization, hydrogenolysis and methanol conversion [97]. It is a black hexagonal crystal with metallic luster, and its hardness is similar to diamond. Tungsten carbide is a good conductor of electricity, is insoluble in water, hydrochloric acid and sulfuric acid, and is easily soluble in a mix of nitric acid and hydrofluoric acid. The first serious discussions and analyses of tungsten carbide emerged during the 2000s with Venkataraman et al. [98] preparing Pt-Ru-W_2_C as a kind of ternary catalyst. The study found that Pt-Ru-W_2_C oxidizes CO at a lower potential, and the polarization for oxidation of hydrogen-containing CO was lower than the widely used Pt-Ru catalysts. At low polarization, the Pt-Ru-W_2_C catalysts showed twice the activity of the Pt-Ru catalysts when the oxidation currents were normalized to the Pt area. The above research shows that tungsten carbide has a good application prospect in the field of catalysis.

In tungsten carbide catalysts, tungsten carbide usually exists in two forms. One is used as a catalytic active component, and the other is used as a support. When tungsten carbides work as a support, they not only provide a pioneering research idea for the construction of metal alloy catalytic active centers, they also open up a promising new way for the further development of high-efficiency catalysts.

In the first form, there is a large volume of published studies describing the role of tungsten carbides working as catalytic active components [13,47]. The research shows that the existence of tungsten carbide increases the density of hydrogen-activating sites and hence enhances the catalytic activity. Commonly used support materials are biochar, carbon nanotubes, MOFs, and so on. The most representative study was performed by Xu et al. [99]. They prepared ultrafine tungsten carbide nanoparticles (with an average size of about 2 nm) by pyrolysis with an RHO-type zeolitic metal azolate framework MAF-6 as the confinement template and carboxyl tungsten as the guest. When the material was used as HER, in 0.5 M H_2_SO_4_, the overpotential was 51 mV, and the Tafel slope was 49 mV/dec under the current density of 10 mA/cm^2^. In terms of exchange current density, its value was twice that of commercial Pt/C. At the same time, the catalysts showed excellent stability and anti-aggregation after a long electrolytic process. The excellent catalytic performance was attributed to the ultrasmall size of tungsten carbides and to the synergy of graphene shells. In principle, this kind of strategy may be extended to synthesize other NPs/nanoclusters for substituting noble metal catalysts.

On this basis, Li and et al. [100] constructed a catalyst composed of N-doped carbon hybridized with a bimetallic molybdenum–tungsten carbide (Mo_X_W_2-X_C) to form compound nanowires for HER. Under the action of tungsten carbides, catalysts obtain an outstanding conductivity and a thermodynamically favorable hydrogen-adsorption free energy (ΔG_H*_). The catalysts they prepared achieved a low overpotential (at the current density of 10 mA/cm^2^) of 115 and 108 mV and a small Tafel slope of 58.5 and 55.4 mV/dec in acidic and alkaline environments, respectively, which can maintain a stable performance for 40 h. These studies provide theoretical and experimental bases for tungsten carbide as a catalytic active component and promote it to obtain further development.

In the second form, the first study on the application of tungsten carbide as a support in catalytic materials was performed by Sheng et al. [101]. In their work, they used ammonium metatungstate (AMT), which was treated by spay drying as a precursor, and the WC catalysts with meso-porosity were prepared by a gas–solid reaction in an atmosphere of CH_4_/H_2_. The WC-supported nanoPt catalysts were prepared by impregnation. The results showed that Pt/WC exhibited an attractive catalytic activity in HER with parameter α in the Tafel equation being 0.292 V, which was between Pt (0.31 V) and Pd (0.53 V). The exchange current density of Pt/WC was 4.42 mA/cm^2^, which is on the same order of magnitude as Pt. This research opens up a new field of tungsten carbide as a support, promoting the development of tungsten carbide catalysts.

In order to further explore the characteristics of tungsten carbide as a supporter, Chhina et al. [102] studied the differences between Pt-loaded WC and Pt-loaded carbon material. The results concluded that WC as a supporter has better stability, but the catalytic activity is relatively poor compared with carbon as the supporter. Since oxidation often exists in the environment, Chhina et al. further explored the catalytic activity of two types of materials after oxidation. The results showed that the catalytic activity of any material decreased, but the catalytic activity of materials with WC as a supporter decreased less. Based on this, we can conclude that WC has great potential as a supporter material, which can realize the catalysts with high load and small size at the same time, which provides guiding significance for future research.

On this basis, Li et al. [103] provided a clear guide for well-controlled growth of model WC_X_ catalysts and applied them to a monatomic field. They used tungsten carbides as the atomic carriers of transition metals Fe and Ni. Breakthrough research has been made regarding monatomic OER catalysts. Tungsten carbide is a new material that is different from the traditional porous carbon monoatomic support. Tungsten carbides can strengthen the complexation of the support to metal ions; conversely, the existence of tungsten oxide clusters can use tungsten carbide as a weak coordination supporter of the monatomic catalytic center in the process of carbonization. Due to the unique structure of tungsten carbide, the atomically dispersed FeNi site has a weak binding force with W and C atoms on the surface as well as high mobility. Therefore, the construction of metal monatomic OER catalysts is realized. The overpotential of the catalysts at 10 mA/cm^2^ is 237 mV. When the catalyst load increases, the overpotential can be further reduced to 211 mV. This achieves an ultra-high mass activity (33.5 A/mg FeNi, at η = 300 mV overpotential) and a catalytic conversion (4.96 s^−1^, at η = 300 mV overpotential). The catalysts also hold ultra-high catalytic stability, and there is no obvious activity attenuation after continuous operation for 1000 h. The structural characterization of the sample after the reaction shows that a stable atomic FeNi oxide layer will be gradually formed on the surface of the material in the OER catalytic process, which protects the support tungsten carbide and maintains high OER activity. This study confirmed that WC_X_ supports constitute a new and promising approach to developing transition metal-based and atomically dispersed catalysts for many electrocatalytic reactions.

Although there are many research studies on the application of tungsten carbide in catalysts, there are still hindrances in its development to a certain extent. With the deepening research on tungsten carbide, researchers have found that the formation temperature of W_2_C and WC compounds is uncertain. When the components of the catalysts are not understood enough, the characterization of them is meaningless. Therefore, Yan et al. [46] used the carbothermal reduction method to explore the formation temperature and formation conditions of various tungsten carbides and prepared tungsten carbide nanoparticles. The research shows that (Figure 7b): tungsten nanoparticles are generated at 800 °C, W_2_C particles are generated at 850 °C, and WC is generated at 1000 °C. In the research process, it was also found that the longer the carbonization reduction time, the smaller and more uniform the particles (Figure 7c–h), which provides guidance for the preparation of tungsten carbide nanomaterials and their nanocompounds. Óvári et al. [104] found that the Mo_2_C-type catalysts can be prepared and can form during hydrocarbon reactions. W, which has similar chemical properties to Mo, may also produce W_2_C under the same conditions: a W_2_C overlayer with a homogeneous C distribution down on W by repeating C_2_H_2_ adsorption. This method may directly produce W_2_C without WC. The above research can clarify the components of tungsten carbide and find the formula of tungsten carbide with the best catalytic activity, which provides application prospects for future research. When discussing the similar properties of W_2_C and Mo_2_C, another problem that cannot be ignored is that tungsten carbide (similar to Mo_2_C) may cause the cyclization of hydrocarbon [105]. How to solve this problem is also for future research.

### 4.2. Tungsten Nitride

The theoretical model of tungsten nitride is not as common as that of tungsten carbide. Through theoretical derivation, it was found that [106] after the sp valence electrons of N are combined with the spd band of W, tungsten nitride is similar to noble metals in structure and chemical reactivity, which comes from the changes in the lattice parameter and electronic properties caused by the N atoms [107,108]. Therefore, tungsten nitride has become an important research direction in the field of catalysis because of its excellent electrical conductivity. It is a brown cubic crystal with hard texture, which is soluble in water and organic solvents.

Tungsten nitrides for catalysis include W_2_N and WN. W_2_N is usually prepared by heating tungsten oxide (WO_3_) and is used in block or particle structures. Wang et al. [109] first explored the formation temperature of W_2_N through the programmed temperature rise method in 1995. The results show that the temperature range from WO_3_ to W_2_N is 773~973 K, which opens up a new opportunity to research tungsten nitrides. Much of the current literature on W_2_N pays particular attention to ORR, and the literature on WN focuses on HER and OER.

The earliest research on W_2_N was carried out by Ma et al. [110], in which they explored the relationship between the W_2_N structure cell coefficient and catalytic performance. The research shows that the smaller the cell coefficient, the better the catalytic performance. However, due to the constraints of the preparation and process, the development of W_2_N is still limited. W_2_N did not continue to develop until Villaseca et al. [111] invented nitration. They used WO_3_ and H_2_WO_4_ as precursors to prepare W_2_N in a N_2_ atmosphere, because N_2_ will produce special gas in the furnace and react with raw materials to form W_2_N. It was found that W_2_N is produced at 773~973 K. This result is the same as in previous studies, which proves the accuracy of the temperature range, and the pure phase is obtained at 875 K. After comparing the W_2_N prepared by two different precursors, it was found that the W_2_N with H_2_WO_4_ as a precursor has a smaller particle size and better catalytic performance. On this basis, Podila et al. [112] prepared W_2_N by using citric acid. This kind of catalyst achieved a morphology with high porosity, can be widely used in the production of H_2_ from NH_3_, and has good catalytic performance, which results in higher activity compared to other tungsten nitride catalysts.

In addition to W_2_N zero-dimensional materials, researchers have studied W_2_N two-dimensional materials. The most representative study is the research performed by Xie et al. [113]. The catalysts obtained by loading W_2_N onto graphene nanorods (GO) has good ORR performance and better catalytic effects than WO_3_/GO catalysts. The ORR’s onset potential at WO_3_/GO is around 0.6 V, whereas that of W_2_N/GO shifted positively to 0.71 V, in which the limiting diffusion current at 0.35 V is 1.4 times stronger than that of WO_3_/GO. The above studies render W_2_N as a potential nonprecious-metal ORR catalyst in many electrocatalysis applications.

In addition, a large and growing body of literature has investigated the use of WN in the field of catalysis. The most representative work is performed by Zhang et al. [114]. They designed a WN-doped porous carbon (nitrogen doped carbon) for ORR. Benefiting from the doping of WN, it increased the amount of more exposed active sites. In O_2_-saturated 0.1 M KOH solutions, the results indicate that the doping of WN showed the best activity in terms of the most positive half-wave potential (−0.099 V), which was better than that of without doping of WN (−0.295 V) and was close to that of commercial Pt/C (−0.097 V) (Figure 7i). The result shows that WN has an efficient catalytical performance for ORR, which gives a theoretical basis for full research.

On this basis, Lv et al. [115] designed triple-function electrocatalysts, which is a coating of a WN nanowire core with a Ni(OH)_2_ shell supported on a carbon fiber paper (WN-Ni(OH)_2_). In LSV curves, WN-Ni(OH)_2_ exhibits better high activity toward HER ($\eta_{20}$ of 170 mV, $\eta_{100}$ of 245 mV) than that of WN ($\eta_{20}$ of 313 mV) and Ni(OH)_2_ ($\eta_{20}$ of 529 mV). However, Pt/C still presents the best high activity toward HER, with $\eta_{20}$ of 55 mV and $\eta_{100}$ of 137 mV. Meanwhile, Pt/C presents with a Tafel slope of 45 mV/dec. The values of the Tafel slope are 96, 118, and 110 mV/dec for WN-Ni(OH)_2_, WN, and Ni(OH)_2_. Lv et al. also tested the OER performance; in 1 M KOH, the overpotentials of WN-Ni(OH)_2_ required to drive 100 and 200 mA/cm^2^ are 330 and 368 mV (Figure 7j), which is far lower than that of Ni(OH)_2_ ($\eta_{100}$ = 358 mV, $\eta_{200}$ = 417 mV) and WN ($\eta_{100}$,$\eta_{200}$ > 400 mV) (Figure 7k), indicating the enhanced water oxidation of WN-Ni(OH)_2_. The prominent performance of WN-Ni(OH)_2_ makes it among the best investigated nitride-based OER catalysts. In summary, Lv et al. synthesized a WN-Ni(OH)_2_ to act as a promising alkaline bifunctional catalyst. These results offer promising bifunctional catalysts for water electrolysis and a hopeful pathway for the performance enhancement strategy of WN catalysts.

In the field of tungsten nitride catalysts, although there have been many studies and the formation temperatures of two kinds of tungsten nitrides are clear, in specific experiments, the temperature of formation for W_2_N and WN also change with different experimental methods and experimental conditions. Therefore, meticulous high-temperature nitridation syntheses and inadequate anisotropy are becoming problems to hinder the development of tungsten nitrides [116]. To solve these problems, in future research, scholars should pay more attention to the specific formation temperature of W_2_N and WN and change the crystal surface exposed by the catalysts through strategic regulation, to control the crystal growth of W_2_N and WN nanostructures and to preserve electrochemical stability while enhancing catalyst activity. Furthermore, exploring the effects of W_2_N and WN compound catalysts on catalytic activity is also a relatively new research area.

### 4.3. Tungsten Oxide

Tungsten oxide as a potential candidate, endowed with a variable crystalline phase, adjustable composition, excellent corrosion resistance in an acid electrolyte, and weak oxygen binding, is also a kind of commonly used W-based nanocatalyst. It is a light yellow triclinic powder crystal. WO_3_ is the most stable of tungsten oxides. It is insoluble in water and inorganic acids except for hydrofluoric acid. When the temperature is higher than 650 °C, it can be reduced by H_2_, and at 1000~1100 °C, it can be reduced by carbon to obtain tungsten powder.

WO_3_ can not only be used in common electrolytic water catalysts, it can also show behaviors suitable for surface reactions and catalysis, such as light-stimulated reactions under visible light [117], which provides a new opportunity for the use of WO_3_ in photocatalytic applications. WO_3_ has also been studied by many scholars in the application of low-chain alkanes [118,119]. It was found that WO_3_ has the selectivity of decarbonylation and decarboxylation [120], which plays a guiding role for subsequent research. The Schottky barrier is easy to form at the interface between a material–support electrode and a material–electrolyte, due to the poor conductivity of tungsten carbide, resulting in higher overpotential required for the reaction [121]. Therefore, when preparing tungsten oxide catalysts, researchers usually use conductive substrates to improve their electrical conductivity, such as nickel foam, carbon cloth, MOFs, etc. In recent years, it has been found that the oxygen vacancy strategy can inspire electrocatalytic activity because it has a positive effect on improving the charge transfer and compensating for the weak hydrogen adsorption of the tungsten trioxide [122]. Therefore, tungsten oxide has invoked significant interest for its unique properties and has been widely studying.

In the field of water splitting, for the above reasons, Lv [123] chose foamed nickel as a substrate, making WO_3-X_/NF nanosheets and WO_3_/NF nanosheets to compare their HER performances. The results show that the catalytic performance of WO_3-X_/NF is higher than that of WO_3_/NF. Specifically, WO_3-X_/NF needs only 175 and 260 mV to reach current densities of 10 and 50 mA/cm^2^, respectively. In addition, the initial potential required for WO_3-X_/NF is only about 100 mV. Another indicator, the Tafel slope, shows that the Tafel slope of WO_3-X_/NF is 110 mV/dec, which is relatively microscopic compared with WO_3_/NF (140 mV/dec) and NF (146 mV/dec), indicating that WO_3-X_/NF has good catalytic reaction kinetics to accelerate the generation of hydrogen. Although compared with Pt/C, there is still a certain gap, through further regulation and preparation, it is believed that they can be comparable to a precious metal base. In this work, it is evidenced that oxygen vacancy defects in tungsten oxide can promote the speed of charge transfer and charge separation and can improve the catalytic performance.

On this basis, Lu et al. [122] further explored and investigated the relationships between different contents of oxygen vacancies and catalytic activities. Lu showed that increasing annealing temperature and the time to obtain a high performance is available. The substoichiometric WO_X_ with more oxygen vacancies exhibits higher HER activity. The current densities at the same potential are proportional to the content of oxygen vacancies, suggesting that these vacancies can boost the kinetics for catalytic reactions. The best WO_X_/C shows that the overpotential at the current density of 10 mA/cm^2^ is 202.60 mV. However, the overpotential at the current density of 10 mA/cm^2^ for Pt/C is 26.61 mV.

WO_3_ can also be used as a support to increase the utilization of low-chain alkanes. Szabó et al. [124] demonstrated that Pt nanoparticles over WO_3_ nanowires in ethanol decomposition reactions show outstanding activity and selectivity. However, the number of studies in this area is still limited. Therefore, there is still a long way to go for tungsten oxides for use in HER.

Except for WO_3_, WO is regarded as a high-performance electrocatalyst, owing to its high charge carrier density, cost-effectiveness and excellent stability. Zhao et al. [125] studied Ru-WO regarding electrocatalytic performance for HER. Ru-WO is a three-dimensional structure that shows an onset overpotential of 32 mV in the LSV curve and a Tafel slope of 34 mV/dev. Moreover, Ru-WO demonstrates an excellent operation stability and a steady current density up to 500 mA/cm^2^ over 50 h. This work provides a promising strategy for the future development of tungsten oxide catalysts for HER.

Because of strong acid and weak O-binding in tungsten oxide, Lu et al. [126], by constructing W and Ir dual sites, achieved a tradeoff on the O-binding properties in OER. They prepared a class of three-dimensional nanocatalysts: Ir-W as the metal core and Ir-WO_3_ as the shell. The surface area of this kind of intermediate is larger than that of the one-dimensional material, and it can also inhibit the formation of peroxides. The overpotential of Ir-W at Ir-WO_3-X_ toward OER catalysis at a current density of 10 mA/cm^2^ is only 261 mV, which is lower than that of Ir-WO_3_ (310 mV) and IrO_2_ (486 mV). In this work, Lu et al. demonstrated that the existence of oxygen vacancies and three-dimensionality can improve catalytic performance to a certain extent. This work can provide significant insights for the design of tungsten oxide catalysts for water splitting.

In recent years, the relatively novel research was performed by Zhang et al. [127]. They prepared psesudocubic phase tungsten oxide as a photocatalyst for HER. In their work, through alternate band structure and active site configuration to produce defect and engineer phases, oxygen vacancies were induced in WO_3_ at a relatively low temperature, accompanying the crystal structure transition from a monoclinic to orthorhombic or to pseudocubic phase. The results demonstrate that the photocatalysts that can host the highest amount of oxygen vacancies affords the highest photocatalytic H_2_ production rate of 340 μmol g^−1^ h^−1^. This may be the first example of controlling the amount of oxygen vacancies to improve the performance of photocatalysts for HER.

Although tungsten oxide is widely studied in the field of catalysts, there are still some problems such as undesirable electronic conductivity and less active sites. Tungsten oxide still has awhile to go before practical application. To break through the limited electrocatalytic performance, more fundamental principles should be focused on, such as controlling the morphology from one-dimensions to three-dimensions.

### 4.4. Tungsten Sulfide

In addition to the tungsten compounds mentioned above, some other tungsten compounds have attracted the attention of researchers. The tungsten sulfide system is the most representative, including tungsten sulfide and tungsten selenide.

Since Hinnemann et al. [128] first found that MoS_2_ has a similar catalytic performance to Pt-based catalysts, two-dimensional sulfide has always been the research hot spot of amorphous nanocatalysts. W is a kind of transition metal, and the structure of WS_2_ is similar to MoS_2_; thus, it has also attracted the attention of more and more scholars. Bonde et al. [129] found that MoS_2_ and WS_2_ showed almost the same activity. Transition metal sulfides are connected by the van der Waals force of S-W-S, and the band gap can be regulated; thus, they are promising catalysts for HER. According to the lamellar structure, there are many shapes of WS_2_, including hexagon, star, tie and triangle, among which triangle is the most common shape (Figure 7l) [130]. According to relevant research findings [128], the catalytic activity of WS_2_ mainly comes from the edge position of the structure, which especially depends on the S atom that provides guidance for the catalytic activity of WS_2_ later.

The most representative research on WS_2_ as an electrocatalyst is the WS_2_ thin film prepared by Matteo et al. [66] using pulse laser deposition (PLD) combined with heat treatment. PLD is a method that is performed by laser ablation of a target material to provide uniform coverage. PLD can be deposited at room temperature, which helps to explore the impact of the material structure. The results show that when the annealing temperature is 500 °C, the starting potential of this kind of catalyst is lower, and the catalytic performance is close to that of Pt. This may be because WS_2_ has a high-density metal edge [131]. It was confirmed by Voiry et al. [132], in which they found that the more the metal phase changes, the greater the state density of the Fermi level and the more enhanced the catalytic activity.

On this basis, Kim et al. [133] further studied WS_2_ grown on W/Si in the case of atomic layer deposition (ALD). In this method, the reactant (tris(hexyne) tungsten monocarbonyl (W(CO)(CH_3_CH_2_C≡CCH_2_CH_3_)_3_) and H_2_S were fed in an alternating pulsed manner separated by the purging of inert gas. In the LSV curve, annealing at 600 °C achieved a current density of 5 mA/cm^2^ at an overpotential of 374 mV. However, the HER performance showed a significant increase for the 700 °C annealed WS_x_. A current density of 10 mA/cm^2^ was achieved at a relatively low overpotential of 282 mV. This improvement in HER was mainly due to the increased enhancement in the crystallinity of the sample. The study revealed the importance of sulfur content and crystallinity on the HER performance of W-based sulfides. In addition, the above research also showed that the presence of the oxide phase in the tungsten sulfide catalyst reduced the catalytic activity, where a significant amount of oxygen can negatively impact performance [134], which provides a theoretical direction for the development of a tungsten sulfide catalyst.

Furthermore, An et al. [67] studied the effect of doping Me atoms (Cr, Mn, Co) on WS_2_. The calculation shows that doping Me atoms will change the electronic state near the Fermi level, making the electron transfer rate faster and the catalytic activity better. Therefore, loading is a method to improve the catalytic activity of WS_2_. These studies provide new opportunities for the research of W-based two-dimensional materials as electrocatalysts.

Based on the study of tungsten oxide and tungsten sulfide, researchers have focused on tungsten selenide, which is a similar sulfur compound. Selenium (Se) and S are in the same main group and different cycles in the periodic table. The outermost layer has six electrons. The chemical properties of tungsten sulfide and tungsten selenide are both similar and different. The atomic radius of Se is larger than S, the ionization energy is less than S, and the metallicity is greater than S. Therefore, the conductivity of selenide is greater than that of sulfide, which will help to improve the catalytic activity of the material.

In recent years, researchers have made many attempts to research tungsten selenide as a hydrogen evolution catalyst. Tsai et al. [135] found that the catalytic active sites of WSe_2_ were mainly concentrated on the edges of Se through the periodic density pan function, which was similar to tungsten oxide and tungsten sulfide. Therefore, the main way to optimize the catalytic hydrogen evolution performance of tungsten selenide is to change the material structure and improve the utilization efficiency of marginal active sites. The main approaches include: (1) preparing single-layer or few-layer WSe_2_ nanoparticles to improve the edge active sites, such as stripping technology; (2) selecting excellent carriers, such that WSe_2_ is grown on the surface of excellent electronic conductors to improve the electronic conductivity.

Romanov et al. [136] demonstrated that the layers stack one by one via the van der Waals force and expose mostly the basal plane with a small portion of edge sites, which is not favorable for electrochemical HER. Thus, they prepared thin films of WSe_2_, which were obtained by using a method of shadow-masked PLD. The reduction current density reached 10 mA/cm^2^ at an overpotential around 245 mV. Although it had lower activity than nanocrystalline, it is still an interesting work that first reported that WSe_X_ could be a great electrocatalyst for HER.

On this basis, Wang et al. [137] synthesized few-layered WSe_2_ nanoflowers anchored on graphene nanosheets through a facile one-pot solvothermal method. In this way, not only was the catalyst conductivity improved, the exposed edge sites were as well, which lead to an enhancement of the charge transfer kinetics between the catalytic edge sites and the graphene nanosheets. Compared with bare WSe_2_, RGO/WSe_2_ demonstrates a highly effective and stable electrocatalytic performance. RGO/WSe_2_ delivers a microscopic Tafel slope of 57.6 mV/dec, a remarkably low onset potential of 150 mV, a high current density of 38.43 mA/cm^2^ at 300 mV and excellent long-term stability. This work could provide significant insights for the design of tungsten selenide catalysts for water splitting.

A large and growing body of literature has investigated chalcogenide tungsten catalysts. It can be concluded that forming compounds, changing material morphology and obtaining more surface defects are effective methods to enhance the catalytic activity of chalcogenide tungsten catalysts.

### 4.5. Summary

Electrochemical parameters of tungsten-based compound nanocatalysts currently reported has been exhibited in Table 4. In the future, the electrochemical method will become one of the most important ways to obtain sustainable energy. At present, nanomaterials and nanocompounds formed by transition metals and nonmetallic elements are considered to be effective electrocatalytic hydrogen evolution catalysts because of their high chemical stability, good electronic conductivity and low resistivity. A growing body of research backs up the argument that the type of tungsten compounds, i.e., different crystal structures, will greatly affect catalytic performance. Inspired by this, crystal engineering strategies involving the target morphology, specific crystal phase and orientation, the local atomic structure as well as charge densities [138,139] are widely concerned by scholars.

## 5. Perspectives for Future Development of Tungsten-Based Nanocatalysts

With the increasing consumption of Pt resources in recent years, it is important to choose a catalyst that can replace commercial Pt/C. Among the many kinds of catalysts that can be selected, W, as a kind of transition metal, has a wide-application prospect. In addition, W is an environmentally friendly material and a potential research object. In terms of existing research, tungsten has a strong Brönsted acid site and excellent water resistance [140], which can be used as a catalyst in electrolytic water. In addition, other types of catalysts retain different advantages; thus, it is expected that there will be various practical application prospects in the future. Tungsten-based catalysts have many advantages. First, W has a variety of oxidation states from −2 to +6; these compounds have diversity in structure, and they are innocuous. Thus, it has great potential in the field of catalysis. Second, more and more countries have mined W, which reduces the price of W. However, the relationship between the structure and performance of tungsten-based catalysts still needs to be solved. Scientific researchers are still in the exploratory stage of the activity, stability and real reaction mechanism of tungsten-based catalysts. How to solve the relationship between the structure and performance of tungsten-based catalysts and then guide and design the catalysts is important for future research and development. This poses a challenge to the potential application of tungsten-based catalysts.

### 5.1. Research Direction of Tungsten-Based Catalysts in the Future

#### 5.1.1. Developing Stable Single Tungsten Atom Catalysts

**Figure 8:**
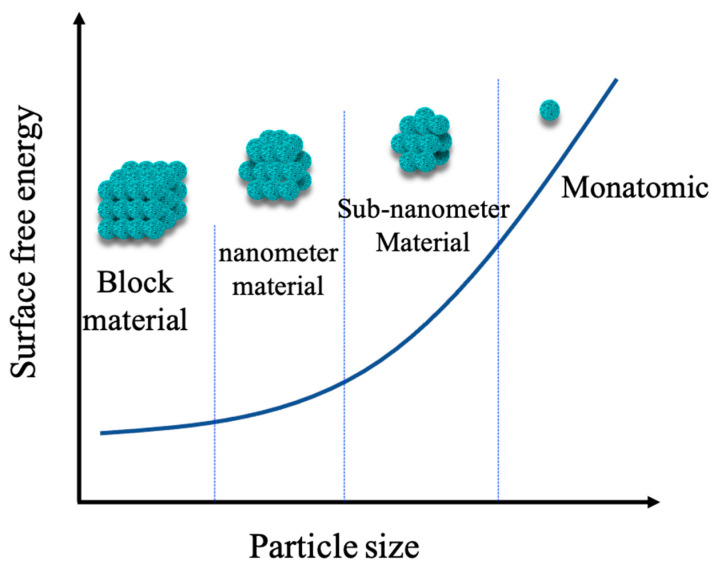
Relationship between particle size and surface free energy.

Theoretically, the dispersion limit of supported catalysts is evenly distributed on the support in the form of a metal single atom. At this time, each atom of a metal catalyst has been fully utilized, and each individual atom is an independent active site. The energy level structure and electronic structure of the particles will face huge changes, such as rapidly increasing surface free energy, quantum size effect, interaction with the metal–support, etc. (Figure 8), which will improve the catalytic efficiency. Therefore, monatomic catalysts have better catalytic activity or selectivity than nanoparticles and sub nanoclusters.

Due to the unique structure of a single atom, single W atom catalysts often show different activity, selectivity and stability from W-based nanocatalysts. Single W atom catalysts can not only reduce the use of W, but can also improve the utilization efficiency of W. Therefore, the vigorous development of a single W atom catalyst is of great significance for the utilization of energy. However, although many remarkable achievements have been made in the field of SACs in recent years [141,142], most of them are carried out in the laboratory, which limits the application of SAC. This is mainly because SAC only has one type of active center, which makes it difficult to break the linear scale relationship [143] between the adsorption energy of reaction intermediates. Therefore, it is necessary to overcome the relationship between particle size and surface adsorption energy before application.

#### 5.1.2. Designing High-performance Tungsten-based Nanocatalysts

The activity of W-based nanocatalysts is usually affected by the conductivity, surface morphology and active sites of materials. Between the conductivity of W itself and the good chemical stability of nanomaterials formed by W and nonmetallic elements, W-based catalysts will become one of the important ways to obtain sustainable energy in the future. Therefore, how to improve the catalytic performance of W-based nanocatalysts has become an urgent problem to be solved. In the laboratory, different researchers have different loading qualities and preparation methods of catalysts on the electrode, resulting in deviation. Therefore, we should formulate a unified standard test to screen the best catalysts for application more accurately. At the same time, in order to find the best electrocatalysts, there are still many problems to be solved: (1) The particle size of an effective catalyst material should be evenly distributed. (2) Compared with zero-dimensional, one-dimensional and two-dimensional structures, three-dimensional structures exhibit the most active sites, that is, high crystallinity materials are the best for catalysts. (3) In order to reduce costs, the catalysts that can be applied to two or more catalytic reactions at the same time have the highest benefit.

In addition, with the application of W-based catalysts, the use of W will increase exponentially in the next 10 years. Therefore, we must pay attention to the recycling of W resources and realize the concept of “green environmental protection”.

### 5.2. Theoretical Calculations for Tungsten-Based Catalysts

#### 5.2.1. DFT Calculations Supporting the Improvement of Catalyst Performance

DFT is a theory for solving single electron problems. At the beginning of the 20th century, researchers established quantum mechanics to describe the objective laws of the micro world. It was found that the motion of microparticles followed the Schrodinger equation. However, the key to the successful solution of the Schrodinger equation is that there is no interaction, but in the actual micro world, we face the interacting multi-particle system. Therefore, researchers simplify the multi-particle system and realize the development from a multi-electron system to a single electron system (Hatree Fock method), which makes it possible to solve the Schrodinger equation.

DFT is developed on the Schrodinger equation. Kohn and Sham proposed to replace the interacting functions in the Schrodinger equation with a noninteracting function and then to add an exchange correlation function to the equation to eliminate all errors. In this way, the kinetic energy and potential energy calculated theoretically can be brought into the equation, and the difference between theory and practice can be offset by exchanging correlation functions. The obtained energy value is the accurate value. Using the modern electronic structure method based on DFT, the quantum chemical calculation of small clusters at two adjacent metal positions in the W compound can be carried out, which can effectively understand the micromorphology on a nano scale, and which has a far-reaching impact on the effective design of reasonable W-based catalysts.

#### 5.2.2. In-Depth Understanding of the Catalytic Reaction Mechanism

No matter what kind of catalysis, its essence is to reduce the reaction activation energy and speed up the reaction process. For electrocatalysis, more theories and experiments are needed to deeply understand the electrochemical reaction mechanism and the changes at the cathode and anode electrodes. By understanding the reaction mechanism, we can simulate the changes of chemical properties in the middle of the reaction and effectively improve the stability of W-based catalysts. In the future, the study of transport mechanisms and kinetics of W ions under different reaction conditions can lay a foundation for the wider application of W-based catalysts.

### 5.3. Preparation Methods of Catalysts

The preparation methods of catalysts have great influence on the shape of catalysts. The usual preparation methods include precipitation, impregnation, the hydrothermal method, the sol-gel method, and so on. However, regardless of the kind of preparation method, the catalysts should preferably be subject to reduction pretreatment, which is generally carried out at the last step of the preparation of W-based catalysts in order to activate the catalysts and effectively avoid reoxidation due to contact with air. This step can effectively improve the stability of the catalysts and can lay a foundation for the preparation of efficient and stable W-based catalysts.

### 5.4. Optimizing Design on Catalysts Supports for Applications

Although current researchers have performed a lot of research on W-based catalysts, W-based catalysts still do not meet the commercial development in terms of stability and catalytic activity. As one of the important components of catalysts, the support material plays a key role in the particle size, catalytic performance, stability and even service life of catalysts. Generally, the ideal catalyst support material should have the following characteristics: (1) high conductivity; (2) large specific surface area and ability to load more and more atoms evenly; (3) stable physical and chemical properties; (4) low price and high safety. Hu et al. [34] found that the combination of the transition metal atom and the carbon material gives the transition metal catalysts excellent catalytic properties. Carbon nanomaterials have a special layered porous structure. When used as a catalyst support, they can provide channels for the transportation of electrolyte ions and electrons, as well as rich active sites for atoms [144]. These advantages give the catalysts good stability and catalytic activity. Therefore, choosing carbon as the support for W-based catalysts is a promising research direction.

### 5.5. Improving Catalytic Activity

In the future, the field of catalysis will become indispensable in research regarding obtaining sustainable energy. In the laboratory, different researchers have different loading qualities and preparation methods of catalysts on electrodes, resulting in deviation. Therefore, we should formulate a unified standard test to screen for the best catalysts for more accurate application. At the same time, in order to find the best catalysts, there are still many problems to be solved: (1) The particle size of the effective catalyst material should be evenly distributed. (2) High crystallinity materials have the best effect on catalysts. (3) In order to reduce costs, the catalysts that can be applied to two or more catalytic reactions at the same time have the highest benefit. It is believed that in the near future, with the improvement in theory and the progress in technology, tungsten-based catalysts will be applied in various catalytic fields to improve daily life.

## Figures and Tables

**Table 1:** Electrochemical parameter of single tungsten atom catalysts currently reported.

Support	Reaction	Overpotential (mV) at Current Density (mA/cm^2^)	Tafel Slope (mV/dec)	Ref.
N-doped carbon	HER	85 at 10 (0.1 M KOH) 105 at 10 (0.5 M H_2_SO_4_)	53 (1 M KOH) 58 (0.5 M H_2_SO_4_)	[15]
ZIFs	ORR	-	71 (KOH)	[32]
GO	NRR	-	8.35% at −0.70 V	[33]
CoP	HER	40 at 10 (1.0 M KOH) 48 at 10 (0.5 M H_2_SO_4_)	47 (1 M KOH) 56 (0.5 M H_2_SO_4_)	[34]
NiS_0.5_Se_0.5_	HER	39 at 10 (1.0 M KOH)	51 (1 M KOH)	[35]
NiS_0.5_Se_0.5_	OER	171 at 10 (1.0 M KOH)	41 (1 M KOH)	[35]

**Table 2:** Electrochemical parameter of the Co–W–Se catalysts for the ORR in 0.5 M H_2_SO_4_.

W Molar Ratio/%	OCP/V Versus SHE	α	−b/V	$j_{0}\times 10^{6}$ $/mA\;cm^{-2}$
0.00	0.79	0.49	0.120	1.58
0.74	0.80	0.49	0.121	2.85
1.49	0.81	0.49	0.120	2.82
2.38	0.79	0.51	0.116	0.66
2.55	0.79	0.45	0.132	2.21

**Table 3:** Electrochemical parameters of tungsten metal nanocatalysts currently reported.

Materials	Reaction	Overpotential (mV) at Current Density (mA/cm^2^)	Tafel Slope (mV/dec)	Ref.
Pt_2_CuW_0.25_/C	ORR	Half-wave potential (929 mV)	55.8 (KOH)	[16]
CoW(OH)_x_	HER	73.6 at 10 (0.1 M PBS) 114.9 at 20 (0.1 MPBS)	149 (0.1 M PBS)	[59]
MoS_2_-W	HER	500 at 124 (0.5 M H_2_SO_4_)	-	[69]
a-WNP	HER	Onset potential (~50 mV) (0.5 M H_2_SO_4_)	39 (0.5 M H_2_SO_4_)	[72]
W:CoO	OER	320 at 10 (pH = 13)	45 (pH = 13)	[74]
MnSi-W	OER	-	109.38 (pH = 7)	[78]
W-CoP	HER	32 at 10 (0.5 M H_2_SO_4_) 77 at 10 (1.0 M KOH)	57 (0.5 M H_2_SO_4_) 65 (1.0 M KOH)	[81]
W-CoP	OER	252 at 10 (1.0 M KOH)	74 (1.0 M KOH)	[81]
Ni-Fe-W LDH	OER	230 at 50 (1.0 M KOH)	64 (1.0 M KOH)	[82]

**Table 4:** Electrochemical parameters of tungsten-based compound nanocatalysts currently reported.

Materials	Reaction	Overpotential (mV) at Current Density (mA/cm^2^)	Tafel Slope (mV/dec)	Ref.
WC at NPC	HER	51 at 10 (0.5 M H_2_SO_4_)	49 (0.5 M H_2_SO_4_)	[99]
Mo_X_W_2-X_C at N-Doped carbon	HER	115 at 10 (0.5 M H_2_SO_4_) 108 at 10 (1.0 M KOH)	58.5 (0.5 M H_2_SO_4_) 55.4 (1.0 M KOH)	[100]
Pt/WC	HER	-	a = 0.292 V b = 0.124 V	[101]
FeNi at WC_X_	OER	211 at 10 (1.0 M KOH)	-	[103]
W_2_N/GO	ORR	120 at 72.96 (1.0 M KOH)	-	[113]
WO_3_/GO	ORR	120 at 27.39 (1.0 M KOH)	-	[113]
WN/Porous carbon	ORR	Half-wave potential (99 mV)	-	[114]
WN	HER	313 at 20 (1.0 M KOH)	118 (1.0 M KOH)	[115]
WN-Ni(OH)_2_	HER	170 at 20 (1.0 M KOH) 245 at 100 (1.0 M KOH)	96 (1.0 M KOH)	[115]
WN	OER	>400 at 100 (1.0 M KOH)	-	[115]
WN-Ni(OH)_2_	OER	330 at 100 (1.0 M KOH) 368 at 200 (1.0 M KOH)	-	[115]
WO_X_	HER	202.6 at 10 (0.5 M H_2_SO_4_)	-	[122]
WO_3-X_/NF	HER	175 at 10 (0.5 M H_2_SO_4_) 260 at 50 (0.5 M H_2_SO_4_)	110 (0.5 M H_2_SO_4_)	[123]
Ru-WO	HER	32 at 0.5 (0.5 M H_2_SO_4_)	34 (0.5 M H_2_SO_4_)	[125]
Ir-WO_3-X_	OER	310 at 10 (1.0 M KOH)	-	[126]
WS_X_	HER	282 at 10 (0.5 M H_2_SO_4_)	-	[133]
WSe_X_	HER	245 at 10 (0.5 M H_2_SO_4_)	-	[136]
RGO/WSe_X_	HER	300 at 38.43 (0.5 M H_2_SO_4_)	57.6 (0.5 M H_2_SO_4_)	[137]

## Data Availability

Not applicable.
